# Whole-volume apparent diffusion coefficient histogram analysis for prediction of programmed cell death ligand 1 expression in periampullary carcinomas: a preliminary study

**DOI:** 10.3389/fonc.2026.1783525

**Published:** 2026-04-29

**Authors:** Lei Bi, Qun Xie, Juntao Zhang, Shifeng Cai, Ximing Wang, Xiaodong Li

**Affiliations:** 1 Department of Radiology, Shandong Provincial Hospital Affiliated to Shandong First Medical University, Jinan, China; 2 Department of Radiology, Women & Children’s Health Care Hospital of Linyi, Linyi, China; 3 Department of Medical Affairs for Pharmaceutical Diagnostics, GE Healthcare, Shanghai, China; 4 Department of Radiology, Linyi People’s Hospital, Linyi, China

**Keywords:** apparent diffusion coefficient, diffusion weighted imaging, histogram analysis, periampullary carcinoma, programmed cell death ligand 1

## Abstract

**Purpose:**

To assess the possibility of employing whole-volume ADC histogram analysis for predicting programmed cell death ligand 1 (PD-L1) expression in periampullary carcinomas (PCs).

**Materials and methods:**

We retrospectively evaluated imaging records of 65 patients with PC who received pancreaticoduodenectomy in our hospital. PD-L1 expression was systematically categorized as positive or negative based on the tumor proportion score (TPS), immune cell score (ICS), and the combined positive score (CPS), with an immunohistochemistry assay. Univariate analysis was conducted to assess differences in parameters between PD-L1-positive and PD-L1-negative groups. Spearman’s correlation analysis was utilized to explore associations between variables and PD-L1 expression. Receiver operating characteristic (ROC) analysis was performed to evaluate the differential diagnostic performance of parameters in distinguishing two groups.

**Results:**

Several ADC histogram parameters were significantly different between PD-L1-positive and PD-L1-negative group, and showed significant correlations with PD-L1 expression, most notably the 5th and 10th percentiles. In TPS grouping, the 5th percentile demonstrated the highest area under the curve (AUC) of 0.690, which was improved to 0.740 when combined with tumor size and carbohydrate antigen 19-9. In ICS grouping, the 10th percentile showed the highest AUC of 0.690, which was improved to 0.772 when integrated with the degree of differentiation. In CPS grouping, the 5th percentile demonstrated the highest AUC of 0.694, which was improved to 0.752 when combined with tumor size and carcinoembryonic antigen.

**Conclusion:**

Whole-volume ADC histogram parameters of primary tumors hold great potential in predicting PD-L1 expression in PCs.

## 1 Introduction

Periampullary carcinoma (PC), a relatively rare malignancy, emerges proximate to the major duodenal papilla (1). Recent trends indicate a rising incidence of this carcinoma, with a meager five-year survival rate hovering around 6% (2). Currently, pancreaticoduodenectomy remains the primary treatment method for PCs (3). However, the elevated surgical risks and complication rates make it challenging to determine the necessity of surgery (4). Therefore, the adoption of novel therapeutic approaches to enhance patient prognosis is crucial.

Programmed cell death ligand 1 (PD-L1), increasingly prevalent in human cancers, has garnered significant scientific interest (5, 6). Recent research has revealed cancer cells’ propensity to overexpress immune inhibitory proteins, particularly in the tumor microenvironment, enabling them to elude host immunity (7). The interaction between PD-L1 and PD-1 on activated T cells curtails T cell proliferation and activity, consequently dampening anti-tumor immunity (8). PD-L1, identified in non-small cell lung cancer, has emerged as a critical biomarker in the realm of advanced cancer therapeutics, guiding the use of anti-PD-1/PD-L1 immunotherapy. This expression serves as a pivotal predictive marker in the management and treatment of this cancer type (9). Melanoma, with its immunogenicity and immune cell abundance, has highlighted the significance of targeting the PD-1/PD-L1 axis in dermatological oncology (10). However, the exploration of PD-L1 expression prediction in periampullary carcinomas remains nascent, with limited studies and practical applications still in preliminary phases (11). This burgeoning area of research opens new prospects for academic and clinical advancements (11–15).

Contemporary functional imaging techniques offer enhanced insights into the intricate microstructure of tumors (5). Diffusion-weighted imaging (DWI), which evaluates the Brownian motion of water molecules within tissues, is quantified using the apparent diffusion coefficient (ADC) (5, 16). This metric inversely correlates with tissue cellularity, where areas of restricted diffusion, evident through increased diffusion-weighted signals, correspond to lower ADC values (16, 17). Volumetric ADC histogram analysis augments the precision of voxel distribution assessments across entire lesions, thereby minimizing the subjectivity of region of interest (ROI) selection and bolstering the accuracy of quantitative ADC evaluations (1). However, the utilization of ADC histogram analysis for predicting PD-L1 expression in periampullary carcinomas remains unexplored. Consequently, this study aims to explore the potential link between MRI-derived ADC histogram values and PD-L1 expression levels in PCs, encompassing an evaluation of PD-L1 staining in both tumor and tumor-related immune cells within the tumor microenvironment.

## 2 Materials and methods

### 2.1 Study population

**Figure 1:**
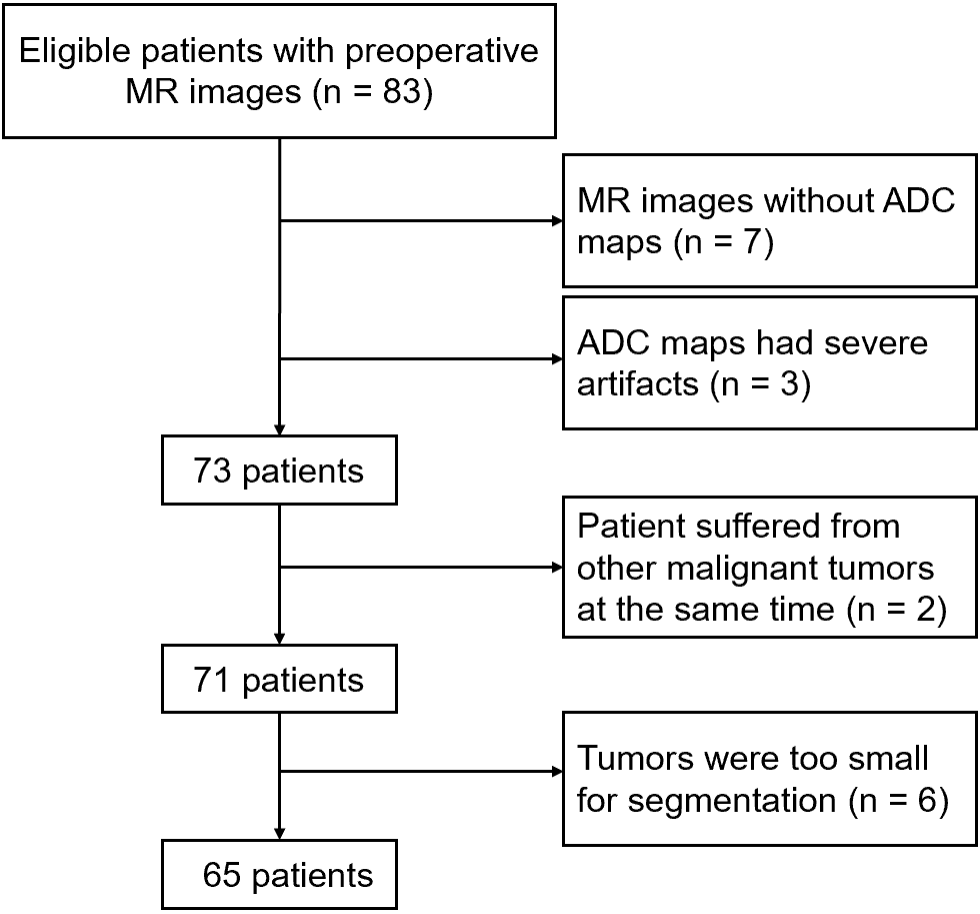
The cohort of patients and study workflow included in the study.

The retrospective analysis of de-identified data in this study received approval from the ethics committee, and obtaining informed consent was waived. We conducted a search in our picture archiving and communication system (PACS) database to identify preoperative MR images of PC patients meeting the following inclusion criteria: 1) underwent curative procedures involving complete tumor removal and lymph node dissection; 2) had extensive clinical and pathological records; 3) received preoperative magnetic resonance imaging within two weeks before surgery; and 4) possessed high-quality ADC maps suitable for segmentation (i.e., free from artifacts). Patients who had undergone radiotherapy or chemotherapy before surgery or had concurrent malignancies were excluded from the study. From June 2012 to September 2019, a total of 83 patients were identified. Among them, seven patients lacked ADC images in their MRI scans, three patients had severe artifacts in their MRI images, and two patients had concurrent tumors. Additionally, in six patients, the tumor volume was too small for accurate segmentation. As a result, the final cohort consisted of 65 patients (**Figure 1**).

### 2.2 MRI parameters

On a 3.0-T MRI system (MAGNETOM Verio, Siemens), MRI exams were carried out utilizing an eight-channel phased-array surface coil. For the DWI sequence, respiratory-triggered, fat-suppressed, single-shot echo-planar imaging on the transverse plane was performed. Each acquisition was obtained using b values of 0 and 800 s/mm2. Depending on the patient’s breathing performance, the total acquisition time varied from 3 to 6 minutes. A commercially available software workstation system (Syngo Multimodality workspace, Siemens) was used to automatically build the ADC map. The following parameters were used to determine the acquisition settings for the DWI sequence: TR/TE = 4000/73 ms; field of view = 380 mm × 285 mm; flip angle = 90°; matrix = 128 × 78; slice thickness = 5 mm; band width = 2442 Hz/pixel; ETL = 78; and echo space = 0.51 ms.Tumor segmentation and histogram analysis.

**Figure 2:**
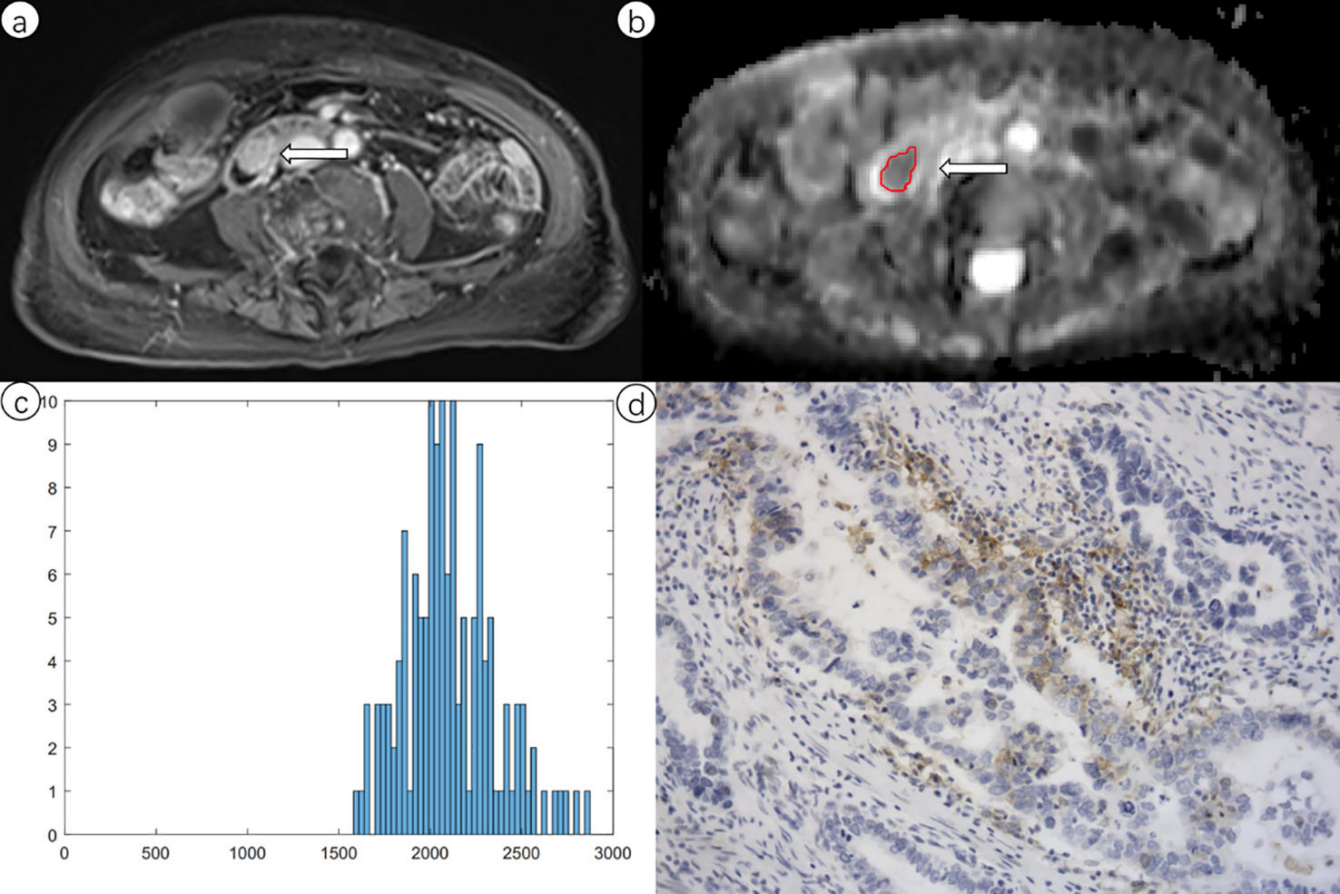
Representative case of periampullary carcinomas in a 65-year-old woman with a positive programmed cell death ligand 1 (PD-L1) score. **(a)** Delayed phase of T1-weighted image reveals marked enhancing tumor of the distal common bile duct (arrow). **(b)** Apparent diffusion coefficient (ADC) map of the same tumor, with a marked region of interest that was manually drawn strictly within the border of the lesion. **(c)** The resulting ADC histogram of the tumor. **(d)** The corresponding PD-L1 stained specimen.

The segmentation process in this study utilized ITK-SNAP (v. 3.8.0) for three-dimensional analysis. Two seasoned radiologists, with respective experiences of 9 and 16 years in abdominal MR interpretation, collaboratively executed the tumor segmentation. To ensure precise analysis of each primary tumor, the region of interest (ROI) was meticulously outlined on every slice of the ADC map, strictly encompassing the primary tumor. This included areas of hematoma, necrosis, or cyst within the tumor region, while ensuring the exclusion of the pancreatic duct, common bile duct, and other adjacent normal structures. Initially, the ROIs were localized on the DWI images, with reference to T1-weighted, T2-weighted, and gadolinium-enhanced images, and then transferred to the ADC maps. Subsequently, the volume of interest (VOI) for each lesion was automatically generated for histogram analysis (**Figure 2**). Feature extraction was performed using PyRadiomics (v. 3.1.0). A range of first-order parameters was obtained, including quartiles (25th, 50th, 75th), 5th, 10th, 90th and 95th percentiles, maximum, minimum, mean ADC values, energy, entropy, kurtosis, range, variance, and skewness.

### 2.3 PD-L1 expression analysis

In this study, PD-L1 expression was retrospectively analyzed using a standard PD-L1 (E1L3N^®^) XP^®^ Rabbit mAb (CST, USA) assay, consistent with commercial guidelines. Specimens from resected tumors were evaluated for PD-L1 expression analysis. Three scoring methods were used: tumor proportion score (TPS), focusing solely on the percentage of positive tumor cells, immune cell score (ICS), focusing solely on the percentage of positive immune related cells, and combined positive score (CPS), encompassing tumor cells and immune related cells. All assessments were conducted at 20x magnification. PD-L1 expression was classified in TPS, ICS and CPS using thresholds of 0, 1% and 5%, respectively. The analysis was performed by a board-certified pathologist, blinded to imaging results.

### 2.4 Statistical analysis

Our study employed a univariate analysis to evaluate disparities in factors between PD-L1-positive and PD-L1-negative cohorts. Categorical variables underwent chi-square, while continuous variables were subjected to Student’s t-test or Mann-Whitney U test. Spearman’s correlation analysis was utilized to explore associations between variables and PD-L1 expression. The analytical capability of various variables to distinguish between two groups was meticulously evaluated through receiver operating characteristic (ROC) curve analysis, employing key metrics such as the area under the curve (AUC), sensitivity, and specificity. The optimal cutoff was ascertained using the Youden index. The inter-observer agreements of all histogram parameters were evaluated with the intraclass correlation coefficient (ICC): 0.00-0.20, poor correlation; 0.21-0.40, fair correlation; 0.41-0.60, moderate correlation; 0.61-0.80, good correlation; and 0.81-1.00, excellent correlation. Statistical analyses were conducted using SPSS and MedCalc software, with significance set at p < 0.05.

## 3 Results

### 3.1 Patient characteristics

Our study encompassed 65 participants. Among them, 21 individuals (11 males, 10 females; mean age, 59.29 years; range, 47–74 years) exhibited PD-L1 positive expression in TPS. Conversely, 44 patients (26 males, 18 females; mean age, 55.39 years; range, 32–78 years) demonstrated PD-L1 negative expression in TPS. Eighteen patients (10 males, 8 females; mean age, 56.22 years; range 39–74 years) were diagnosed with positive PD-L1 expression in ICS, while 47 patients (27 males, 20 females; mean age, 56.81 years; range, 32–78 years) were diagnosed with negative PD-L1 expression in ICS. Furthermore, 16 patients (7 males, 9 females; mean age, 59.31 years; range 47–74 years) were diagnosed with positive PD-L1 expression in CPS, while 49 patients (30 males, 19 females; mean age, 55.78 years; range, 32–78 years) were diagnosed with negative PD-L1 expression in CPS. Among these patients, 14 tumors (21.54%) were located in the duodenum, 10 (15.38%) in the ampulla of Vater, 18 (27.69%) in the common bile duct, and 23 (35.38%) in the pancreas. Thirty-seven tumors (56.92%) were pancreatobiliary-type and 28 tumors (43.08%) were intestinal-type. The tumor sizes ranged from 1.0-6.0 cm, with an average size of 2.85 cm. Within the cohort, pathological examination confirmed lymph node metastasis in 21 cases (32.31%). The histopathological analysis revealed a differentiation spectrum across the tumor samples: 4 (6.15%) were classified as well-differentiated, 40 (61.54%) were moderate differentiation, and 21 (32.31%) were poorly differentiated. Detailed patient and tumor characteristics were comprehensively presented in **Table 1**–**3**.

**Table 1:** Patient and tumor characteristics in TPS grouping.

Characteristic	PD-L1-negative group (n = 44)	PD-L1-positive group (n = 21)	p value
Gender			0.609
Male	26 (59.09)	11 (52.38)	
Female	18 (40.91)	10 (47.62)	
Age (years, mean ± SD)	55.39 ± 10.95	59.29 ± 6.90	0.631
Tumor origin			0.856
Common bile duct	13 (29.55)	5 (23.81)	
Ampulla of Vater	7 (15.91)	3 (14.29)	
Duodenum	10 (22.73)	4 (19.05)	
Pancreas	14 (31.82)	9 (42.86)	
Pathological differentiation			0.575
Intestinal type	20 (45.45)	8 (38.10)	
Pancreatobiliary type	24 (54.55)	13 (61.90)	
Tumor size (cm, mean ± SD)	2.88 ± 1.27	2.79 ± 1.10	0.073
CA125 (U/ml, mean ± SD)	28.72 ± 58.07	19.37 ± 14.76	0.442
CA19-9 (U/ml, mean ± SD)	242.14 ± 308.64	151.73 ± 185.94	0.339
CEA (ng/ml, mean ± SD)	7.87 ± 25.36	9.43 ± 23.77	0.346
LN status			0.491
LNM- negative	31 (70.45)	13 (61.90)	
LNM- positive	13 (29.55)	8 (38.10)	
Degree of differentiation			0.158
High	4 (9.09)	0	
Moderate	24 (54.55)	16 (76.19)	
Low	16 (36.36)	5 (23.81)	

Data are number of patients; data in parentheses are percentage unless otherwise indicated. CA125, carbohydrate antigen 125; CA19-9, carbohydrate antigen 19-9; CEA, carcinoembryonic antigen; LN, lymph node; LNM, lymph node metastasis; SD, standard deviation; TPS, tumor proportion score.

**Table 2:** Patient and tumor characteristics of the ICS grouping.

Characteristic	PD-L1-negative group (n = 47)	PD-L1-positive group (n = 18)	p value
Gender			0.890
Male	27 (57.45)	10 (55.56)	
Female	20 (42.55)	8 (44.44)	
Age (years, mean ± SD)	56.81 ± 10.49	56.22 ± 8.60	0.263
Tumor origin			0.241
Common bile duct	16 (34.04)	2 (11.11)	
Ampulla of Vater	7 (14.89)	3 (16.67)	
Duodenum	8 (17.02)	6 (33.33)	
Pancreas	16 (34.04)	4 (38.89)	
Pathological differentiation			0.069
Intestinal type	17 (36.17)	11 (61.11)	
Pancreatobiliary type	30 (63.83)	7 (38.89)	
Tumor size (cm, mean ± SD)	2.87 ± 1.08	2.78 ± 1.53	0.148
CA125 (U/ml, mean ± SD)	28.25 ± 56.52	19.06 ± 12.33	0.442
CA19-9 (U/ml, mean ± SD)	193.41 ± 268.26	263.90 ± 299.89	0.467
CEA (ng/ml, mean ± SD)	7.60 ± 24.54	10.41 ± 25.68	0.439
LN status			0.483
LNM- negative	33 (70.21)	11 (61.11)	
LNM- positive	14 (29.79)	7 (38.89)	
Degree of differentiation			< 0.001
High	4 (8.51)	0	
Moderate	22 (46.81)	18 (100)	
Low	21 (44.68)	0	

Data are number of patients; data in parentheses are percentage unless otherwise indicated. CA125, carbohydrate antigen 125; CA19-9, carbohydrate antigen 19-9; CEA, carcinoembryonic antigen; LN, lymph node; LNM, lymph node metastasis; SD, standard deviation; ICS, immune cell score.

**Table 3:** Patient and tumor characteristics of the CPS grouping.

Characteristic	PD-L1-negative group (n = 49)	PD-L1-positive group (n = 16)	p value
Gender			0.220
Male	30 (61.22)	7 (43.75)	
Female	19 (38.78)	9 (56.25)	
Age (years, mean ± SD)	55.78 ± 10.57	59.31 ± 7.36	0.219
Tumor origin			0.822
Common bile duct	15 (30.61)	3 (18.75)	
Ampulla of Vater	7 (14.29)	3 (18.75)	
Duodenum	10 (20.41)	4 (25.00)	
Pancreas	17 (34.69)	6 (37.50)	
Pathological differentiation			0.520
Intestinal type	20 (40.82)	8 (50.00)	
Pancreatobiliary type	29 (59.18)	8 (50.0)	
Tumor size (cm, mean ± SD)	2.89 ± 1.23	2.74 ± 1.18	0.673
CA125 (U/ml, mean ± SD)	28.22 ± 56.81	17.26 ± 10.59	0.511
CA19-9 (U/ml, mean ± SD)	231.68 ± 301.79	176.48 ± 205.24	0.500
CEA (ng/ml, mean ± SD)	7.72 ± 24.81	11.77 ± 29.11	0.610
LN status			0.609
LNM- negative	34 (69.39)	10 (62.50)	
LNM- positive	15 (30.61)	6 (37.50)	
Degree of differentiation			0.320
High	4 (8.16)	0	
Moderate	28 (57.14)	12 (75.00)	
Low	17 (34.69)	4 (25.00)	

Data are number of patients; data in parentheses are percentage unless otherwise indicated. CA125, carbohydrate antigen 125; CA19-9, carbohydrate antigen 19-9; CEA, carcinoembryonic antigen; LN, lymph node; LNM, lymph node metastasis; SD, standard deviation; CPS, combined positive score.

### 3.2 Univariate analysis

According to ICC, excellent inter-observer agreement was obtained for all histogram parameters (ICC, 0.875-0.999).

#### 3.2.1 PD-L1 expression of the TPS grouping

Within the TPS categorization, clinical and pathological features did not significantly vary between PD-L1 negative and positive groups (**Table 1**). In the PD-L1-positive group, the 5th percentile (p = 0.014), 10th percentile (p = 0.028), minimum ADC value (p = 0.018), and mean ADC value (p = 0.048) were notably lower than in the PD-L1-negative group. A negative correlation with PD-L1 expression was observed for these parameters: 5th percentile (r = -0.309, p = 0.012), 10th percentile (r = -0.275, p = 0.026), minimum ADC value (r = -0.295, p = 0.017), and mean ADC value (r = -0.247, p = 0.047). Conversely, other parameters did not exhibit significant differences between the groups. Detailed comparisons and correlations are presented in **Table 4**.

**Table 4:** Histogram parameters and ROC results in TPS grouping.

Histogram parameters	PD-L1-negative group	PD-L1-positive group	p value	AUC (95% CI)	Sensitivity (%)	Specificity (%)	Correlation (r)	p value of correlation test
5th percentile (× 10–^6^ mm^2^/sec)	1026.525 (891.938, 1304.100)	892.600 (737.250, 1043.300)	0.014	0.690 (0.552, 0.828)	0.682	0.619	-0.309	0.012
10th percentile (× 10–^6^ mm^2^/sec)	1087.150 (934.650, 1396.275)	956.500 (793.100, 1161.700)	0.028	0.670 (0.525, 0.815)	0.818	0.476	-0.275	0.026
25th percentile (× 10–^6^ mm^2^/sec)	1195.750 (1047.938, 1511.875)	1099.000 (908.250, 1310.250)	0.078	0.636 (0.488, 0.784)	0.909	0.333	-0.220	0.078
50th ADC value (× 10–^6^ mm^2^/sec)	1348.250 (1180.000, 1581.625)	1229.000 (1029.250, 1513.750)	0.125	0.619 (0.467, 0.770)	0.545	0.714	-0.192	0.125
75th percentile (× 10–^6^ mm^2^/sec)	1504.625 (1342.750, 1752.000)	1395.000 (1159.500, 1704.500)	0.090	0.631 (0.473, 0.788)	0.659	0.667	-0.212	0.090
90th percentile (× 10–^6^ mm^2^/sec)	1670.950 (1467.100, 2003.550)	1461.000 (1315.750, 1857.700)	0.090	0.631 (0.476, 0.786)	0.614	0.667	-0.212	0.090
95th percentile (× 10–^6^ mm^2^/sec)	1557.335 ± 405.054	1597.300 (1365.025, 1917.550)	0.110	0.623 (0.471, 0.776)	0.682	0.571	-0.200	0.110
Minimum ADC value (× 10–^6^ mm^2^/sec)	821.500 (607.750, 1149.250)	664.810 ± 313.358	0.018	0.682 (0.548, 0.816)	0.455	0.905	-0.295	0.017
Mean ADC value (× 10–^6^ mm^2^/sec)	1392.469 (1234.230, 1639.187)	1239.590 (1078.533, 1593.409)	0.048	0.653 (0.499, 0.806)	0.614	0.714	-0.247	0.047
Maximum ADC value (× 10–^6^ mm^2^/sec)	2557.910 ± 543.191	2514.430 ± 650.806	0.778	0.517 (0.362, 0.673)	1.000	0.143	-0.028	0.824
Energy (× 10^-10^)	0.804 (0.102, 2.837)	2.126 (0.558, 5.423)	0.123	0.619 (0.477, 0.761)	0.714	0.568	0.193	0.124
Entropy	5.280 (4.679, 5.635)	5.311 ± 0.616	0.280	0.583 (0.434, 0.733)	0.905	0.273	0.135	0.284
Kurtosis	3.699 (2.820, 4.442)	3.826 ± 1.289	0.966	0.503 (0.351, 0.656)	0.810	0.295	0.005	0.967
Range	1630.910 ± 714.172	1849.620 ± 666.619	0.243	0.615 (0.468, 0.762)	0.524	0.750	0.186	0.138
Variance (× 10^-6^)	0.065 (0.044, 0.112)	0.091 ± 0.056	0.483	0.554 (0.406, 0.703)	0.333	0.818	0.088	0.487
Skewness	0.717 (0.258, 1.010)	0.590 ± 0.560	0.613	0.544 (0.395, 0.693)	0.432	0.714	-0.072	0.569

For data conforming to a normal distribution, the mean and standard deviation are used for description; for data not conforming to a normal distribution, the median and interquartile range are used for description. ADC, apparent diffusion coefficient; AUC, area under the curve; CI, confidence interval; PD-L1, programmed cell death ligand 1; ROC, receiver operating characteristic; TPS, tumor proportion score.

#### 3.2.2 PD-L1 expression of the ICS grouping

Within the ICS categorization, PD-L1 positive tumors were all diagnosed as moderately differentiated, which were significantly different between PD-L1 negative group, the other clinical and pathological features did not significantly vary between two groups (**Table 2**). In the PD-L1-positive group, the 5th percentile (p = 0.024), 10th percentile (p = 0.018), and 25th percentile (p = 0.033) were significantly lower compared to those in the PD-L1-negative group. A negative correlation with PD-L1 expression was observed for these parameters: 5th percentile (r = -0.282, p = 0.023), 10th percentile (r = -0.295, p = 0.017) and 25th percentile (r = -0.267, p = 0.032). In the PD-L1-positive group, kurtosis (p = 0.024) was significantly higher compared to the PD-L1-negative group. Kurtosis showed a positive correlation with PD-L1 expression (r = 0.282, p = 0.023). Conversely, other parameters did not exhibit significant differences between the groups. Detailed comparisons and correlations are presented in **Table 5**.

**Table 5:** Histogram parameters and ROC results in ICS grouping.

Histogram parameters	PD-L1-negative group	PD-L1-positive group	p value	AUC (95% CI)	Sensitivity (%)	Specificity (%)	Correlation (r)	p value of correlation test
5th percentile (× 10–^6^ mm^2^/sec)	1020.200 (891.250, 1209.300)	876.450 (718.550, 1095.650)	0.024	0.682 (0.522, 0.842)	0.638	0.722	-0.282	0.023
10th percentile (× 10–^6^ mm^2^/sec)	1070.800 (932.400, 1255.600)	936.650 (787.350, 1113.750)	0.018	0.690 (0.535, 0.846)	0.702	0.667	-0.295	0.017
25th percentile (× 10–^6^ mm^2^/sec)	1206.000 (1044.000, 1426.000)	1080.250 (897.875, 1198.375)	0.033	0.672 (0.517, 0.827)	0.660	0.722	-0.267	0.032
50th ADC value (× 10–^6^ mm^2^/sec)	1373.000 (1167.500, 1556.000)	1230.000 (1016.375, 1341.750)	0.069	0.647 (0.492, 0.802)	0.532	0.778	-0.227	0.069
75th percentile (× 10–^6^ mm^2^/sec)	1506.000 (1317.750, 1803.000)	1379.250 (1189.875, 1549.063)	0.098	0.634 (0.480, 0.787)	0.596	0.667	-0.207	0.098
90th percentile (× 10–^6^ mm^2^/sec)	1636.600 (1447.400, 2034.200)	1542.150 (1401.000, 1797.350)	0.265	0.590 (0.440, 0.740)	0.660	0.556	-0.139	0.269
95th percentile (× 10–^6^ mm^2^/sec)	1835.666 ± 439.996	1747.986 ± 365.867	0.491	0.556 (0.403, 0.708)	0.638	0.556	-0.086	0.495
Minimum ADC value (× 10–^6^ mm^2^/sec)	810.000 (579.000, 1056.000)	653.500 (504.750, 885.750)	0.115	0.627 (0.473, 0.781)	0.596	0.722	-0.197	0.116
Mean ADC value (× 10–^6^ mm^2^/sec)	1382.886 (1221.260, 1639.762)	1274.702 (1075.344, 1428.213)	0.067	0.648 (0.491, 0.804)	0.936	0.333	-0.229	0.066
Maximum ADC value (× 10–^6^ mm^2^/sec)	2503.810 ± 590.311	2648.440 ± 535.971	0.369	0.573 (0.421, 0.724)	0.778	0.426	0.113	0.371
Energy (× 10^-10^)	1.022 (0.073, 2.542)	1.848 (0.628, 6.062)	0.110	0.629 (0.485, 0.772)	0.778	0.468	0.200	0.111
Entropy	5.295 (4.648, 5.654)	5.353 (5.011, 5.664)	0.509	0.553 (0.409, 0.697)	1.000	0.234	0.082	0.514
Kurtosis	3.294 (2.686, 4.355)	4.257 (3.462, 5.092)	0.024	0.682 (0.540, 0.824)	0.833	0.532	0.282	0.023
Range	1608.170 ± 704.189	1945.440 ± 651.165	0.083	0.628 (0.480, 0.776)	0.611	0.617	0.199	0.112
Variance (× 10^-6^)	0.065 (0.044, 0.115)	0.071 (0.049, 0.123)	0.608	0.541 (0.395, 0.687)	1.000	0.191	0.064	0.612
Skewness	0.571 ± 0.593	0.833 ± 0.543	0.109	0.665 (0.511, 0.820)	0.444	0.894	0.257	0.039

For data conforming to a normal distribution, the mean and standard deviation are used for description; for data not conforming to a normal distribution, the median and interquartile range are used for description. ADC, apparent diffusion coefficient; AUC, area under the curve; CI, confidence interval; ICS, immune cell score; PD-L1, programmed cell death ligand 1; ROC, receiver operating characteristic.

#### 3.2.3 PD-L1 expression of the CPS grouping

Within the CPS categorization, clinical and pathological features did not significantly vary between PD-L1 negative and positive groups (**Table 3**). In the PD-L1-positive group, the 5th percentile (p = 0.021), 10th percentile (p = 0.028), and mean ADC value (p = 0.031) were significantly lower compared to those in the PD-L1-negative group. A negative correlation with PD-L1 expression was observed for these parameters: 5th percentile (r = -0.289, p = 0.019), 10th percentile (r = -0.274, p = 0.027) and mean ADC value (r = -0.270, p = 0.029). Conversely, other parameters did not exhibit significant differences between the groups. Detailed comparisons and correlations are presented in **Table 6**.

**Table 6:** Histogram parameters and ROC results in CPS grouping.

Histogram parameters	PD-L1-negative group	PD-L1-positive group	p value	AUC (95% CI)	Sensitivity (%)	Specificity (%)	Correlation (r)	p value of correlation test
5th percentile (× 10–^6^ mm^2^/sec)	1020.200 (886.350, 1297.400)	871.950 (726.513, 1053.500)	0.021	0.694 (0.549, 0.839)	0.625	0.714	-0.289	0.019
10th percentile (× 10–^6^ mm^2^/sec)	1070.800 (930.950, 1367.850)	936.650 (797.950, 1076.550)	0.028	0.684 (0.534, 0.834)	0.812	0.490	-0.274	0.027
25th percentile (× 10–^6^ mm^2^/sec)	1185.500 (1042.750, 1509.250)	1085.500 (907.125, 1244.625)	0.062	0.656 (0.503, 0.809)	0.625	0.653	-0.233	0.062
50th ADC value (× 10–^6^ mm^2^/sec)	1323.500 (1173.250, 1575.750)	1217.500 (1021.250, 1433.875)	0.076	0.649 (0.485, 0.812)	0.750	0.531	-0.222	0.076
75th percentile (× 10–^6^ mm^2^/sec)	1503.250 (1338.125, 1784.500)	1366.875 (1148.875, 1601.188)	0.061	0.657 (0.484, 0.830)	0.438	0.918	-0.234	0.060
90th percentile (× 10–^6^ mm^2^/sec)	1637.500 (1460.100, 1986.900)	1444.950 (1302.975, 1781.650)	0.065	0.654 (0.484, 0.825)	0.563	0.796	-0.230	0.065
95th percentile (× 10–^6^ mm^2^/sec)	1854.089 ± 409.894	1680.606 ± 436.153	0.153	0.644 (0.477, 0.811)	0.625	0.674	-0.215	0.085
Minimum ADC value (× 10–^6^ mm^2^/sec)	807.000 (573.500, 1144.500)	660.500 (538.250, 840.000)	0.094	0.640 (0.501, 0.780)	0.938	0.429	-0.209	0.094
Mean ADC value (× 10–^6^ mm^2^/sec)	1382.886 (1226.963, 1646.864)	1232.629 (1072.815, 1510.783)	0.031	0.681 (0.516, 0.846)	0.375	0.980	-0.270	0.029
Maximum ADC value (× 10–^6^ mm^2^/sec)	2589.163 ± 544.737	2405.125 ± 659.966	0.270	0.588 (0.416, 0.760)	0.625	0.592	-0.131	0.297
Energy (× 10^-10^)	1.022 (0.133, 2.964)	2.043 (0.439, 5.911)	0.322	0.583 (0.422, 0.744)	0.688	0.531	0.124	0.326
Entropy	5.312 (4.833, 5.645)	5.160 (4.925, 5.808)	0.867	0.514 (0.337, 0.691)	0.313	0.857	0.021	0.869
Kurtosis	3.702 (2.862, 4.730)	3.417 (2.695, 4.759)	0.784	0.523 (0.345, 0.701)	0.563	0.592	-0.034	0.786
Range	1685.245 ± 704.473	1751.563 ± 712.964	0.746	0.541 (0.373, 0.709)	0.438	0.714	0.061	0.630
Variance (× 10^-6^)	0.071 (0.046, 0.115)	0.065 (0.045, 0.130)	0.939	0.506 (0.334, 0.679)	0.625	0.531	-0.010	0.940
Skewness	0.666 ± 0.580	0.576 ± 0.623	0.601	0.545 (0.371, 0.718)	0.625	0.571	-0.067	0.598

For data conforming to a normal distribution, the mean and standard deviation are used for description; for data not conforming to a normal distribution, the median and interquartile range are used for description. ADC, apparent diffusion coefficient; AUC, area under the curve; CI, confidence interval; CPS, combined positive score; PD-L1, programmed cell death ligand 1; ROC, receiver operating characteristic.

### 3.3 ROC analysis

#### 3.3.1 PD-L1 expression of the TPS grouping

The AUC of 0.690 (95% confidence interval [CI]: 0.552, 0.828) of the 5th percentile exhibited the greatest ability to distinguish between the PD-L1-positive and PD-L1-negative groups, with a sensitivity of 68.2% and a specificity of 61.9%. Followed by the 10th percentile, which had an AUC of 0.670 (95% CI: 0.525, 0.815), with a sensitivity of 81.8% and a specificity of 47.6%. For the minimum ADC value, the AUC was 0.682 (95% CI: 0.548, 0.816), with a sensitivity of 45.5%, and a specificity of 90.5%. Furthermore, the mean ADC recorded an AUC of 0.653 (95% CI: 0.499, 0.806), with a sensitivity of 61.4% and a specificity of 71.4%.

**Figure 3:**
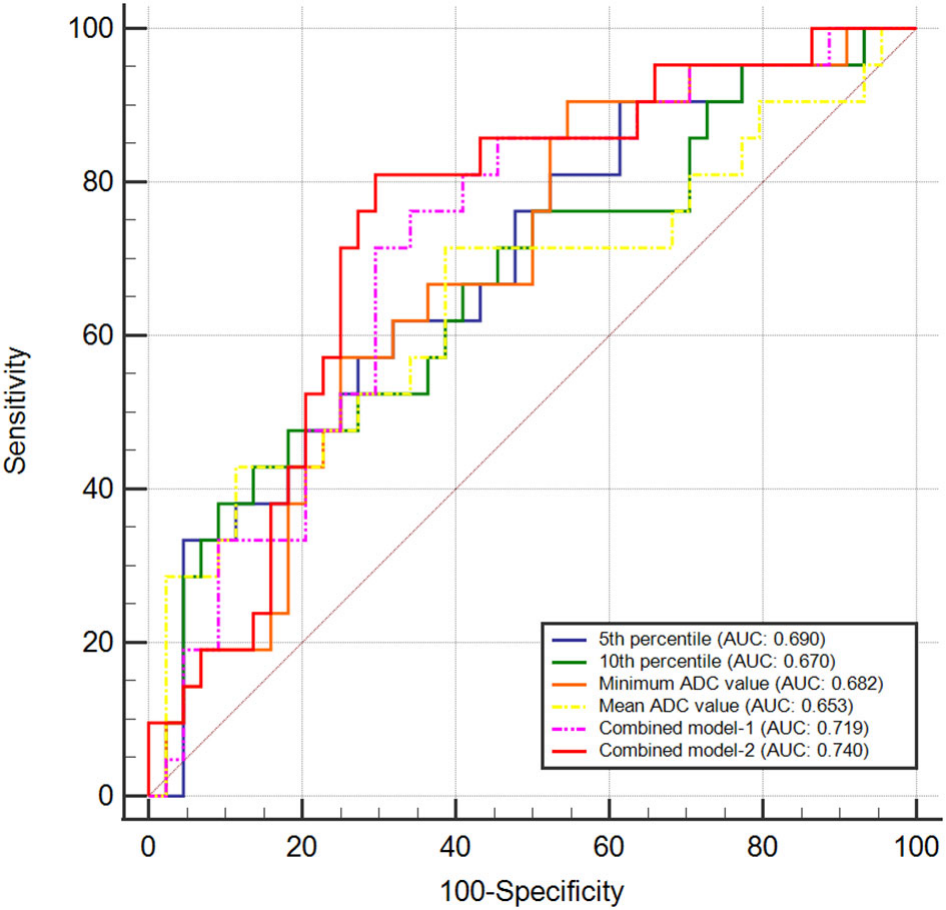
Receiver operating characteristic curves evaluating diagnostic performance in differentiating PD-L1-positive from PD-L1-negative groups using TPS categorization. Combined model-1 incorporated 5th percentile and CA19-9. Combined model-2 incorporated 5th percentile, tumor size and CA19-9.

When analyzing the diagnostic predictive values using a combined model that included carbohydrate antigen 19-9 (CA19-9) and the 5th percentile, the AUC increased to 0.719 (95% CI: 0.589, 0.848), with a sensitivity of 76.2% and specificity of 65.9%. Incorporating tumor size, CA19–9 levels, and the 5th percentile yielded optimal diagnostic efficacy (AUC = 0.740, 95% CI: 0.614, 0.867), demonstrating a sensitivity of 81.0% and a specificity of 70.5%. The ROC curve analyses are shown in **Figure 3**, and the diagnostic performance of the parameters is presented in **Table 4**.

#### 3.3.2 PD-L1 expression of the ICS grouping

The AUC of 0.690 (95% CI: 0.535, 0.846) of the 10th percentile exhibited the greatest ability to distinguish between the PD-L1-positive and PD-L1-negative groups, with a sensitivity of 70.2% and a specificity of 66.7%. Followed by the 5th percentile showed the highest AUC of 0.682 (95% CI: 0.522, 0.842), with a sensitivity of 63.8%, and a specificity of 72.2%. For the 25th percentile, the AUC was 0.672 (95% CI: 0.517, 0.827), with a sensitivity of 66.0%, and a specificity of 72.2%. Furthermore, the kurtosis recorded an AUC of 0.682 (95% CI: 0.540, 0.824), with a sensitivity of 83.3% and a specificity of 53.2%.

**Figure 4:**
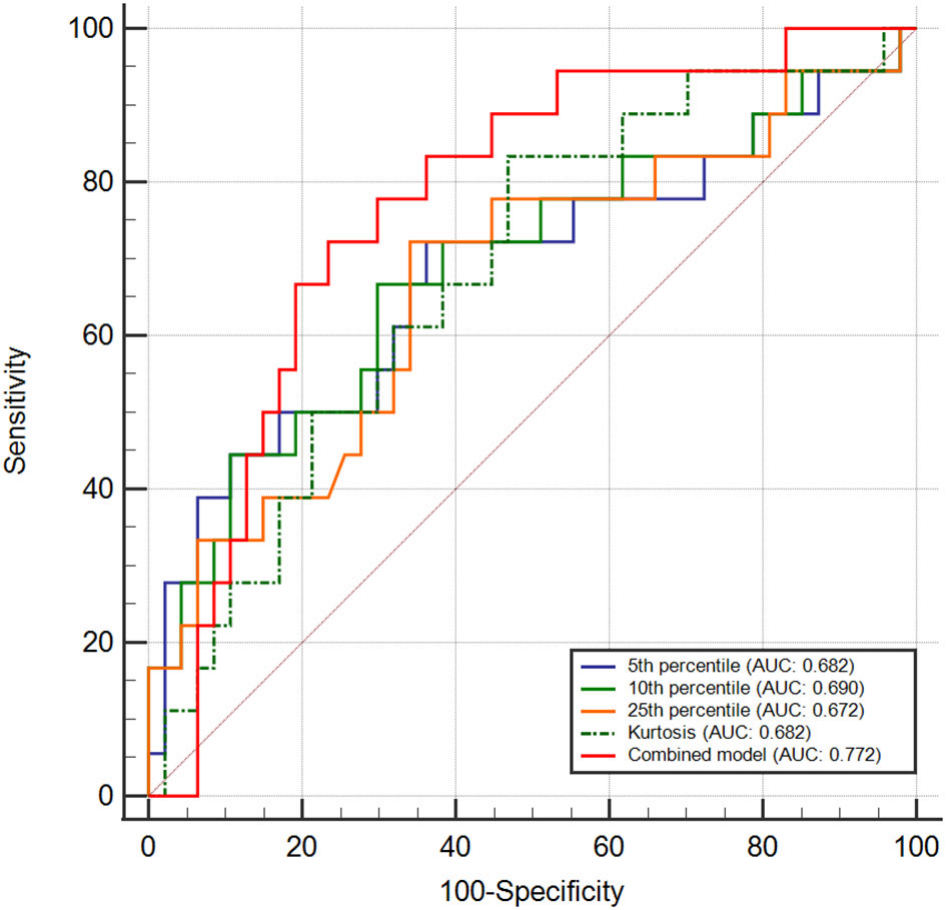
Receiver operating characteristic curves evaluating diagnostic performance in differentiating PD-L1-positive from PD-L1-negative groups using ICS categorization. Combined model incorporated 10th percentile and degree of differentiation.

When analyzing the diagnostic predictive values using a combined model that included the degree of differentiation and 10th percentile, the AUC increased to 0.772 (95% CI: 0.649, 0.894), with a sensitivity of 72.2% and specificity of 76.6%. The ROC curve analyses are shown in **Figure 4**, and the diagnostic performance of the parameters is presented in **Table 5**.

#### 3.3.3 PD-L1 expression of the CPS grouping

The AUC of 0.694 (95% CI: 0.549, 0.839) of the 5th percentile exhibited the greatest ability to distinguish between the PD-L1-positive and PD-L1-negative groups, with a sensitivity of 62.5% and a specificity of 71.4%. Followed by the 10th percentile showed the highest AUC of 0.684 (95% CI: 0.534, 0.834), with a sensitivity of 81.2%, and a specificity of 49.0%. For the mean ADC value, the AUC was 0.681 (95% CI: 0.516, 0.846), with a sensitivity of 37.5%, and a specificity of 98.0%.

**Figure 5:**
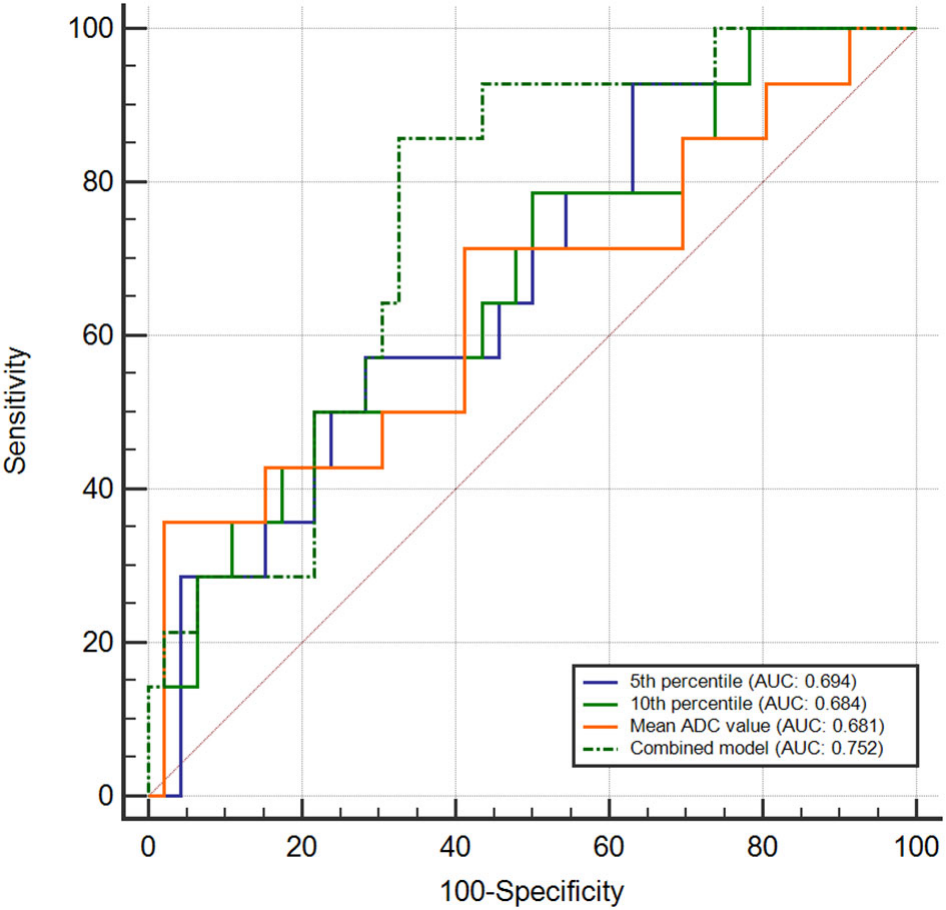
Receiver operating characteristic curves evaluating diagnostic performance in differentiating PD-L1-positive from PD-L1-negative groups using CPS categorization. Combined model incorporated 5th percentile, tumor size and CEA.

When analyzing the diagnostic predictive values using a combined model that included the tumor size, carcinoembryonic antigen (CEA) levels, and the 5th percentile, the AUC increased to 0.752 (95% CI: 0.617, 0.886), with a sensitivity of 85.7% and specificity of 67.4%. The ROC curve analyses are shown in **Figure 5**, and the diagnostic performance of the parameters is presented in **Table 6**.

### 3.4 Subgroup analysis based on histological subtypes

Since pathological differentiation into pancreatobiliary-type and intestinal-type could significantly influence ADC values and patient progrosis of periampullary carcinomas, we conducted subgroup analysis based on histological subtypes.

#### 3.4.1 PD-L1 expression of the intestinal-type

Within the ICS categorization, the 5th percentile (p = 0.029), 10th percentile (p = 0.029), 25th percentile (p = 0.029), and minimum ADC value (p = 0.048) in the PD-L1-positive group were significantly lower compared to those in the PD-L1-negative group. A negative correlation with PD-L1 expression was observed for these parameters: 5th percentile (r = -0.421, p = 0.026), 25th percentile (r = -0.421, p = 0.026), and minimum ADC value (r = -0.385, p = 0.043). In the PD-L1-positive group, energy (p = 0.047) was significantly higher compared to the PD-L1-negative group. Energy showed a positive correlation with PD-L1 expression (r = 0.385, p = 0.043). Conversely, other parameters did not exhibit significant differences between the groups (**Table 7**).

**Table 7:** PD-L1 expression of the intestinal type in ICS grouping.

Histogram parameters	PD-L1-negative group	PD-L1-positive group	p value	Correlation (r)	p value of correlation test
5th percentile (× 10–^6^ mm^2^/sec)	994.400 (891.925, 1368.450)	753.400 (692.350, 955.500)	0.029	-0.421	0.026
10th percentile (× 10–^6^ mm^2^/sec)	1103.500 (953.300, 1396.500)	823.800 (778.800, 1022.700)	0.029	-0.421	0.026
25th percentile (× 10–^6^ mm^2^/sec)	1267.500 (1071.625, 1494.625)	986.500 (873.500, 1127.000)	0.029	-0.421	0.026
Minimum ADC value (× 10–^6^ mm^2^/sec)	943.294 ± 439.828	600.273 ± 407.847	0.048	-0.385	0.043
Energy (× 10^-10^)	0.169 (0.067, 1.683)	1.960 (0.346, 6.664)	0.047	0.385	0.043

For data conforming to a normal distribution, the mean and standard deviation are used for description; for data not conforming to a normal distribution, the median and interquartile range are used for description. ADC, apparent diffusion coefficient; ICS, immune cell score; PD-L1, programmed cell death ligand 1.

Within TPS and CPS categorizations, no parameters exhibited significant differences between the groups.

#### 3.4.2 PD-L1 expression of the pancreatobiliary-type

Within TPS categorization, the minimum ADC value in the PD-L1-positive group (612.000 [497.500, 858.500]) were significantly lower compared to those in the PD-L1-negative group (902.500 [642.250, 1102.000]) (p = 0.039). A negative correlation with PD-L1 expression was observed (r = -0.345, p = 0.037).

Within CPS categorization, the 90th percentile in the PD-L1-positive group (1444.950 [1235.750, 1596.600]) were significantly lower compared to those in the PD-L1-negative group (1734.100 [1460.100, 1982.400]) (p = 0.043). A negative correlation with PD-L1 expression was observed (r = -0.338, p = 0.041).

Within ICS categorization, no parameters exhibited significant differences between the groups.

## 4 Discussion

This study investigated the diagnostic value of whole-volume ADC histogram analysis in predicting PD-L1 expression in PC patients. Our findings revealed that several ADC histogram parameters were significantly different between PD-L1-positive and PD-L1-negative group, and showed significant correlations with PD-L1 expression, most notably the 5th and 10th percentiles. In TPS grouping, the 5th percentile demonstrated the highest AUC of 0.690, which was improved to 0.740 when combined with tumor size and CA 19-9. In ICS grouping, the 10th percentile showed the highest AUC of 0.690, which was improved to 0.772 when integrated with the degree of differentiation. In CPS grouping, the 5th percentile demonstrated the highest AUC of 0.694, which was improved to 0.752 when combined with tumor size and CEA.

Recent studies have identified PD-L1 expression in various tumor types, such as non-small cell lung cancer (18), melanoma (19), and in PC (11). Therapies targeting the PD-1/PD-L1 pathway and the use of PD-L1 expression as a prognostic tool have garnered significant attention. PD-L1 is present not only on tumor cells but also on immune cells, including tumor-related macrophages and dendritic cells (20). The binding of PD-1 to its ligand PD-L1 activates signaling pathways that suppress T cell-mediated immune responses, facilitating tumor immune evasion, and consequently, fostering tumor growth and spread (21–23).

This PD-L1 expression was intricately associated with immune evasion within the tumor microenvironment. Elevated levels of PD-L1 expression typically coincide with reduced immune cell infiltration and tumor immune escape (24). Consequently, heightened PD-L1 expression emerges as a key mechanism underpinning tumor immune evasion. Combination therapies have shown effectiveness in inhibiting tumor growth and improving patient prognosis (25). These discoveries have catalyzed the advancement of immune checkpoint inhibitors, designed to obstruct the PD-1/PD-L1 signaling pathway, consequently augmenting T cell-mediated immune responses against tumors (26).

Immunotherapy, while currently showing limited efficiency in the treatment of PCs and predominantly remaining in the realm of laboratory research, has not been broadly applied in clinical settings (27). However, there were notable successful precedents. In independent studies, Vikram Pothuri et al. and Baoshan Wang et al. have each demonstrated a significant clinical observation: PC patients with heightened PD-L1 expression experienced notable tumor shrinkage and a reduction in CEA levels following targeted immunotherapy treatments (12, 28). Therefore, we aimed to use ADC histograms to predict PD-L1 expression levels, and further assist in the facilitation of immunotherapy.

Recent years, the field of radiomics is experiencing swift progress. As a primary feature within this domain, the ADC histogram has demonstrated its utility across an array of tumor types. Previous studies have proposed that ADC values may reflect several factors related to tumor characteristics. Reduced ADC values are often associated with elevated cell density (29, 30). This phenomenon is due to the restriction of water molecule diffusion between cells, and lower ADC values may indicate an increase in intra-tumoral cell density, possibly implying higher cell infiltration-a reflection of tumor biological features (29, 31, 32). Xi Zhong et al. demonstrated that ADC histogram parameters, specifically mean ADC value, minimum ADC value, and maximum ADC value, were reliable and reproducible tools that could potentially be used to predict PD-L1 expression in nasopharyngeal carcinoma (33). Our research findings indicated that in tumors with higher PD-L1 expression, the ADC values were usually lower, which is consistent with previous studies (5, 33, 34). The binding of PD-1 to PD-L1 can induce apoptosis of T cells, allowing tumor cells to evade immune surveillance and thereby promoting tumor proliferation and disease progression. This mechanism may explain the significant correlation observed between higher PD-L1 expression and lower ADC values (34).

In our study, we identified significant correlations between certain histogram parameters and PD-L1 expression in PCs. This finding suggests a potential link between these imaging characteristics and the molecular profile of the tumor, which could have implications for targeted therapies and patient management in clinical settings. However, the majority of parameters showed no significant correlation. Notably, PCs are widely recognized for their pronounced heterogeneity and diverse biological behaviors, encompassing different tumor subtypes: pancreatobiliary-type and intestinal-type, each potentially exhibiting distinct characteristics (35, 36). The observed weak correlation may be attributed to the intrinsic heterogeneity within this diverse cancer entity, suggesting that the relationship between ADC values and PD-L1 expression could potentially diverge among different tumor subtypes. Therefore, we also conducted subgroup analysis based on histological subtypes. Our results showed that in different subtypes, the ADC histogram also revealed significant correlations with PD-L1 expression. The association should be further investigated to explore its clinical significance and potential implications for PC management.

Our research suggests a connection between ADC histogram parameters and PD-L1 expression in PC patients, yet it is crucial to pursue additional studies to confirm this link. There are several limitations to address. First, the retrospective design of our study inherently carries a risk of selection bias. Second, the sample size is comparatively small and there was a lack of external validation. Third, for the convenience of clinical application, each DWI sequence was obtained using b values of 0 and 800 s/mm^2^. The absence of higher b values might have left the ADC measurements susceptible to perfusion-related “pseudo-diffusion” effects.

In conclusion, the whole-volume ADC histogram parameters of primary tumors demonstrate significant promise in forecasting PD-L1 expression in periampullary carcinomas. This could theoretically underpin and enhance immunotherapy approaches for patients with periampullary carcinoma. Further research is warranted to delve into its clinical relevance and the potential impact on patient care management.

## Data Availability

The raw data supporting the conclusions of this article will be made available by the authors, without undue reservation.

## References

[1] Lu JY Yu H Zou XL Li Z Hu XM Shen YQ . Apparent diffusion coefficient-based histogram analysis differentiates histological subtypes of periampullary adenocarcinoma. World J Gastroenterol. (2019) 25:6116–28. doi: 10.3748/wjg.v25.i40.6116. PMID: 31686767 PMC6824280

[2] Lundgren S Elebro J Heby M Nodin B Leandersson K Micke P . Quantitative, qualitative and spatial analysis of lymphocyte infiltration in periampullary and pancreatic adenocarcinoma. Int J Cancer. (2020) 146:3461–73. doi: 10.1002/ijc.32945. PMID: 32129882

[3] Bi L Liu Y Xu J Wang X Zhang T Li K . A CT-based radiomics nomogram for preoperative prediction of lymph node metastasis in periampullary carcinomas. Front Oncol. (2021) 11:632176. doi: 10.3389/fonc.2021.632176. PMID: 34395237 PMC8358686

[4] Zhang Y Duan Z Yu X Zhang Y Liu J Liao S . Defects of endoscopic biopsy in the diagnosis of periampullary carcinoma and recommendations for diagnosis and treatment: a retrospective study before and after surgery. Gland Surg. (2022) 11:1395–403. doi: 10.21037/gs-22-412. PMID: 36082089 PMC9445708

[5] Meyer HJ Höhn AK Surov A . Relationships between apparent diffusion coefficient (ADC) histogram analysis parameters and PD-L1-expression in head and neck squamous cell carcinomas: a preliminary study. Radiol Oncol. (2021) 55:150–7. doi: 10.2478/raon-2021-0005. PMID: 33764703 PMC8042826

[6] Sun C Mezzadra R Schumacher TN . Regulation and function of the PD-L1 checkpoint. Immunity. (2018) 48:434–52. doi: 10.1016/j.immuni.2018.03.014. PMID: 29562194 PMC7116507

[7] Dermani FK Samadi P Rahmani G Kohlan AK Najafi R . PD-1/PD-L1 immune checkpoint: potential target for cancer therapy. J Cell Physiol. (2019) 234:1313–25. doi: 10.1002/jcp.27172. PMID: 30191996

[8] Gou Q Dong C Xu H Khan B Jin J Liu Q . PD-L1 degradation pathway and immunotherapy for cancer. Cell Death Dis. (2020) 11:955. doi: 10.1038/s41419-020-03140-2. PMID: 33159034 PMC7648632

[9] Hsu PC Jablons DM Yang CT You L . Epidermal growth factor receptor (EGFR) pathway, yes-associated protein (YAP) and the regulation of programmed death-ligand 1 (PD-L1) in non-small cell lung cancer (NSCLC). Int J Mol Sci. (2019) 20:3821. doi: 10.3390/ijms20153821. PMID: 31387256 PMC6695603

[10] Xu L Zhang Y Tian K Chen X Zhang R Mu X . Apigenin suppresses PD-L1 expression in melanoma and host dendritic cells to elicit synergistic therapeutic effects. J Exp Clin Cancer Res. (2018) 37:261. doi: 10.1186/s13046-018-0929-6. PMID: 30373602 PMC6206930

[11] Thakur N Paik KY Hwang G Chong Y . High expression of PD-L1 is associated with better survival in pancreatic/periampullary cancers and correlates with epithelial to mesenchymal transition. Diagnostics (Basel). (2021) 11:597. doi: 10.3390/diagnostics11040597. PMID: 33810560 PMC8065840

[12] Wang B Li D Zeng D Wang W Jiang C . Case report: Advanced primary squamous cell carcinoma in the periampullary area with upregulation of programmed cell death-ligand 1 expression and response to sintilimab immunotherapy. Front Immunol. (2023) 14:1086760. doi: 10.3389/fimmu.2023.1086760. PMID: 36776865 PMC9911421

[13] Ahn S Lee Y Kim JW Lee JC Hwang JH Yoon YS . Programmed cell death ligand-1 (PD-L1) expression in extrahepatic biliary tract cancers: a comparative study using 22C3, SP263 and E1L3N anti-PD-L1 antibodies. Histopathology. (2019) 75:526–36. doi: 10.1111/his.13901. PMID: 31081949

[14] Lim YJ Koh J Kim K Chie EK Kim B Lee KB . High ratio of programmed cell death protein 1 (PD-1)(+)/CD8(+) tumor-infiltrating lymphocytes identifies a poor prognostic subset of extrahepatic bile duct cancer undergoing surgery plus adjuvant chemoradiotherapy. Radiother Oncol. (2015) 117:165–70. doi: 10.1016/j.radonc.2015.07.003. PMID: 26235847

[15] Mou H Yu L Liao Q Hou X Wu Y Cui Q . Successful response to the combination of immunotherapy and chemotherapy in cholangiocarcinoma with high tumour mutational burden and PD-L1 expression: a case report. BMC Cancer. (2018) 18:1105. doi: 10.1186/s12885-018-5021-2. PMID: 30419854 PMC6233589

[16] Chavhan GB Alsabban Z Babyn PS . Diffusion-weighted imaging in pediatric body MR imaging: principles, technique, and emerging applications. Radiographics. (2014) 34:E73–88. doi: 10.1148/rg.343135047. PMID: 24819803

[17] Malayeri AA El Khouli RH Zaheer A Jacobs MA Corona-Villalobos CP Kamel IR . Principles and applications of diffusion-weighted imaging in cancer detection, staging, and treatment follow-up. Radiographics. (2011) 31:1773–91. doi: 10.1148/rg.316115515. PMID: 21997994 PMC8996338

[18] Huang MY Jiang XM Wang BL Sun Y Lu JJ . Combination therapy with PD-1/PD-L1 blockade in non-small cell lung cancer: strategies and mechanisms. Pharmacol Ther. (2021) 219:107694. doi: 10.1016/j.pharmthera.2020.107694. PMID: 32980443

[19] Chen G Huang AC Zhang W Zhang G Wu M Xu W . Exosomal PD-L1 contributes to immunosuppression and is associated with anti-PD-1 response. Nature. (2018) 560:382–6. doi: 10.1038/s41586-018-0392-8. PMID: 30089911 PMC6095740

[20] Dammeijer F van Gulijk M Mulder EE Lukkes M Klaase L van den Bosch T . The PD-1/PD-L1-checkpoint restrains T cell immunity in tumor-draining lymph nodes. Cancer Cell. (2020) 38:685–700.e8. doi: 10.1016/j.ccell.2020.09.001. PMID: 33007259

[21] Guo D Tong Y Jiang X Meng Y Jiang H Du L . Aerobic glycolysis promotes tumor immune evasion by hexokinase2-mediated phosphorylation of IκBα. Cell Metab. (2022) 34:1312–1324.e6. doi: 10.1016/j.cmet.2022.08.002. PMID: 36007522

[22] Zhang J Bu X Wang H Zhu Y Geng Y Nihira NT . Cyclin D-CDK4 kinase destabilizes PD-L1 via cullin 3-SPOP to control cancer immune surveillance. Nature. (2019) 553:91–5. doi: 10.1038/nature25015. PMID: 29160310 PMC5754234

[23] Meyer HJ Höhn AK Surov A . Associations between histogram analysis parameters derived from dynamic-contrast enhanced MRI and PD L1-expression in head and neck squamous cell carcinomas. A preliminary study. Magn Reson Imaging. (2020) 72:117–21. doi: 10.1016/j.mri.2020.07.005. PMID: 32663619

[24] Majidpoor J Mortezaee K . The efficacy of PD-1/PD-L1 blockade in cold cancers and future perspectives. Clin Immuno. (2021) 226:108707. doi: 10.1016/j.clim.2021.108707. PMID: 33662590

[25] Zhang X Lao M Xu J Duan Y Yang H Li M . Combination cancer immunotherapy targeting TNFR2 and PD-1/PD-L1 signaling reduces immunosuppressive effects in the microenvironment of pancreatic tumors. J Immunother Cancer. (2022) 10:e003982. doi: 10.1016/j.hpb.2022.05.646. PMID: 35260434 PMC8906048

[26] Shen X Zhao B . Efficacy of PD-1 or PD-L1 inhibitors and PD-L1 expression status in cancer: meta-analysis. BMJ. (2018) 362:k3529. doi: 10.1136/bmj.k3529. PMID: 30201790 PMC6129950

[27] Duan Z Zhang Y Tang Y Gao R Bao J Liang B . Adjuvant therapy for periampullary carcinoma and the significance of histopathological typing: a systematic review. Transl Oncol. (2022) 20:101414. doi: 10.1016/j.tranon.2022.101414. PMID: 35397420 PMC9006738

[28] Pothuri V Herndon J Ballentine SJ Lim KH Fields RC . A case of a pathological complete response to neoadjuvant nivolumab plus ipilimumab in periampullary adenocarcinoma. Oncologist. (2021) 26:722–6. doi: 10.1002/onco.13821. PMID: 33982365 PMC8417855

[29] Chen L Liu M Bao J Xia Y Zhang J Zhang L . The correlation between apparent diffusion coefficient and tumor cellularity in patients: a meta-analysis. PloS One. (2013) 8:e79008. doi: 10.1371/journal.pone.0079008. PMID: 24244402 PMC3823989

[30] Koral K Mathis D Gimi B Gargan L Weprin B Bowers DC . Common pediatric cerebellar tumors: correlation between cell densities and apparent diffusion coefficient metrics. Radiology. (2013) 268:532–7. doi: 10.1148/radiol.13121362. PMID: 23564715

[31] Koh DM Collins DJ . Diffusion-weighted MRI in the body: applications and challenges in oncology. AJR Am J Roentgenol. (2007) 188:1622–35. doi: 10.2214/ajr.06.1403. PMID: 17515386

[32] Liu R Li R Fang J Deng K Chen C Li J . Apparent diffusion coefficient histogram analysis for differentiating solid ovarian tumors. Front Oncol. (2022) 12:904323. doi: 10.3389/fonc.2022.904323. PMID: 35978817 PMC9376384

[33] Zhong X Li L Yin J Chen Y Xin X Yu L . Reproducibility and usefulness of quantitative apparent diffusion coefficient measurements for predicting program death-ligand 1 expression in nasopharyngeal carcinoma. Cancer Imaging. (2023) 23:98. doi: 10.1186/s40644-023-00587-2. PMID: 37828560 PMC10571377

[34] Zhou X Su X Yu L Wang F Yu S Yu F . Evaluation of programmed cell death ligand-1 expression in primary central nervous system lymphoma using whole-tumor histogram analysis of multiparametric MRI: implications for immunotherapy selection. Front Immunol. (2025) 16:1676273. doi: 10.3389/fimmu.2025.1676273. PMID: 41459535 PMC12738945

[35] Bi L Dong Y Jing C Wu Q Xiu J Cai S . Differentiation of pancreatobiliary-type from intestinal-type periampullary carcinomas using 3.0T MRI. J Magn Reson Imaging. (2016) 43:877–86. doi: 10.1002/jmri.25054. PMID: 26395453

[36] Bi L Yang L Ma J Cai S Li L Huang C . Dynamic contract-enhanced CT-based radiomics for differentiation of pancreatobiliary-type and intestinal-type periampullary carcinomas. Clin Radiol. (2022) 77:e75–83. doi: 10.1016/j.crad.2021.09.010. PMID: 34753589

